# Multi-omic approach identifies a transcriptional network coupling innate immune response to proliferation in the blood of COVID-19 cancer patients

**DOI:** 10.1038/s41419-021-04299-y

**Published:** 2021-10-29

**Authors:** Andrea Sacconi, Claudia De Vitis, Luisa de Latouliere, Simona di Martino, Francesca De Nicola, Frauke Goeman, Carla Mottini, Francesca Paolini, Michela D’Ascanio, Alberto Ricci, Agostino Tafuri, Paolo Marchetti, Arianna Di Napoli, Luciano De Biase, Andrea Negro, Christian Napoli, Paolo Anibaldi, Valentina Salvati, Darragh Duffy, Benjamin Terrier, Maurizio Fanciulli, Carlo Capalbo, Salvatore Sciacchitano, Giovanni Blandino, Giulia Piaggio, Rita Mancini, Gennaro Ciliberto

**Affiliations:** 1 UOSD Clinical Trial Center, Biostatistics and Bioinformatics, IRCCS Regina Elena National Cancer Institute, Via Elio Chianesi 53, 00144, Rome, Italy; 2 Department of Clinical and Molecular Medicine, Sant’Andrea Hospital, Sapienza University of Rome, Rome, Italy; 3 UOSD SAFU, IRCCS Regina Elena National Cancer Institute, Via Elio Chianesi 53, 00144 Rome, Italy; 4 UOSD Clinical Pathology, IRCCS Regina Elena National Cancer Institute, Via Elio Chianesi 53, 00144 Rome, Italy; 5 UOSD Tumor Immunology and Immunotherapy, IRCCS Regina Elena National Cancer Institute, Via Elio Chianesi 53, 00144 Rome, Italy; 6 Department of Medical-Surgical Sciences and of Translational Medicine, Sapienza University of Rome, Sant’Andrea Hospital, Rome, Italy; 7 Hospital Direction and Clinical Departments, Sant’Andrea University Hospital, Rome, Italy; 8 Scientific Direction, IRCCS Regina Elena National Cancer Institute, Via Elio Chianesi 53, 00144, Rome, Italy; 9 Institut Pasteur, Laboratory of Dendritic Cell Immunobiology, Department of Immunology, Paris, France; 10 Department of Internal Medicine, National Referral Center for Rare Systemic Autoimmune Diseases, Assistance Publique Hôpitaux de Paris-Centre, University of Paris, Paris, France; 11 Laboratory of Biomedical Research, Niccolò Cusano University Foundation, Rome, Italy; 12 UOSD Oncogenomica ed Epigenetica, IRCCS Regina Elena National Cancer Institute, Via Elio Chianesi 53, 00144 Rome, Italy

**Keywords:** Cancer, Immunology, Infectious diseases

## Abstract

Clinical outcomes of COVID-19 patients are worsened by the presence of co-morbidities, especially cancer leading to elevated mortality rates. SARS-CoV-2 infection is known to alter immune system homeostasis. Whether cancer patients developing COVID-19 present alterations of immune functions which might contribute to worse outcomes have so far been poorly investigated. We conducted a multi-omic analysis of immunological parameters in peripheral blood mononuclear cells (PBMCs) of COVID-19 patients with and without cancer. Healthy donors and SARS-CoV-2-negative cancer patients were also included as controls. At the infection peak, cytokine multiplex analysis of blood samples, cytometry by time of flight (CyTOF) cell population analyses, and Nanostring gene expression using Pancancer array on PBMCs were performed. We found that eight pro-inflammatory factors (IL-6, IL-8, IL-13, IL-1ra, MIP-1a, IP-10) out of 27 analyzed serum cytokines were modulated in COVID-19 patients irrespective of cancer status. Diverse subpopulations of T lymphocytes such as CD8^+^T, CD4^+^T central memory, Mucosal-associated invariant T (MAIT), natural killer (NK), and γδ T cells were reduced, while B plasmablasts were expanded in COVID-19 cancer patients. Our findings illustrate a repertoire of aberrant alterations of gene expression in circulating immune cells of COVID-19 cancer patients. A 19-gene expression signature of PBMCs is able to discriminate COVID-19 patients with and without solid cancers. Gene set enrichment analysis highlights an increased gene expression linked to Interferon α, γ, α/β response and signaling which paired with aberrant cell cycle regulation in cancer patients. Ten out of the 19 genes, validated in a real-world consecutive cohort, were specific of COVID-19 cancer patients independently from different cancer types and stages of the diseases, and useful to stratify patients in a COVID-19 disease severity-manner. We also unveil a transcriptional network involving gene regulators of both inflammation response and proliferation in PBMCs of COVID-19 cancer patients.

## Introduction

The impact of the COVID-19 pandemic on cancer patients has become a major focus of the investigation. The association of COVID-19 and cancer is responsible for a more severe clinical course with worse outcomes and a lethality rate of up to 25% in these patients [1, 2], and the risk of adverse outcomes of SARS-CoV-2 infection is significantly higher for patients with cancer versus those without. The development of cancer itself, surgical treatment and related procedures, radiotherapy, and/or systemic treatments may lead to immune suppression [3], thus increasing the risk of SARS-Cov-2 infection [4–6]. The negative impact of COVID-19 has been seen across a broad spectrum of cancers [7–10]. Although cancer by itself may be a risk factor for the infection and evolution of the COVID-19 disease, the biological mechanisms underlying this synergy have been scarcely investigated.

During SARS-CoV-2 infection, a derailed immune response of the host is the main cause of tissue damage and the resulting severity of the disease. Serum cytokines are aberrantly produced in these patients and circulating subsets of immune cells are altered compared to healthy donors. In agreement, lymphocytes' gene expression profiles are deeply modified in Covid-19 patients and these alterations are further enhanced with disease severity [11–13]. Being lymphopenia, the strongest predictor for severity of disease in COVID-19 patients [14]. An impaired interferon-I (IFN-1) response that leads to a low expression of interferon-stimulated genes coupled with high levels of cytokines represents a hallmark of COVID-19 infection [12, 15–17]. Few data are available about the modulation of immune cells in COVID-19 patients with cancer. A multicentre study reported a significant decrease in the number of T and NK cells in COVID-19 patients with hematological malignancies when compared to COVID-19 patients [18]. Given the key role of the immune response in both COVID-19 and cancer, we hypothesized that the presence of SARS-CoV-2 in a host immunologically compromised by cancer, could result in specific features useful as prognostic markers of disease progression. Based on this hypothesis, our main purpose was to find immunological features specific to cancer patients infected with SARS-CoV-2 and to unveil how COVID-19 disease impacts the immune system of these patients. To this purpose, using a multi-omic experimental approach, we investigated COVID-19 patients affected by neoplasms, serum cytokines, changes in the proportion of circulating subsets of immune cells, and PBMC immunological gene expression profiles. Of note, we identified a gene expression signature, common to different cancer types and independent from stages of the diseases. Moreover, we identified for the first time a network including transcription factors (TFs) known to be relevant for the innate response upon viral infection and cancer chronic inflammation, such as IRF, STAT, and BATF and a master regulator of proliferation, E2F, which underlies aberrant immune and proliferation responses of PBMCs in COVID-19 cancer patients.

## Results

### Patients characteristics

We collected blood samples to perform single-cell and molecular immunological analysis by fCyTOF and NanoString technologies of ten COVID-19 patients and four healthy donors (HD). In five out of ten patients, COVID-19 was associated with cancer. We have also included, in the Nanostring analysis, a cohort of ten patients with cancer without COVID-19. The Nanostring results have been further validated by RT-PCR including additional two cancer patients without COVID-19 and 16 cancer COVID-19 patients (Supplementary Fig. 1). Clinical and epidemiological characteristics of this group of patients are reported in Supplementary Table I. According to the criteria reported by the Chinese National Health Commission [19, 20], we subdivided COVID-19 patients in five critical, eight severe, six mild, three moderate, and four asymptomatic. Among cancer patients, we collected 13 lung cancer, 6 hematological malignancy, 5 gastrointestinal cancer, 2 breast cancer, 2 clear cell renal cell carcinoma, and 5 other malignancies (Supplementary Fig. 1 and Supplementary Table I).

### Symptomatic COVID-19 instigates hypercytokinemia irrespective of cancer status

**Fig. 1:**
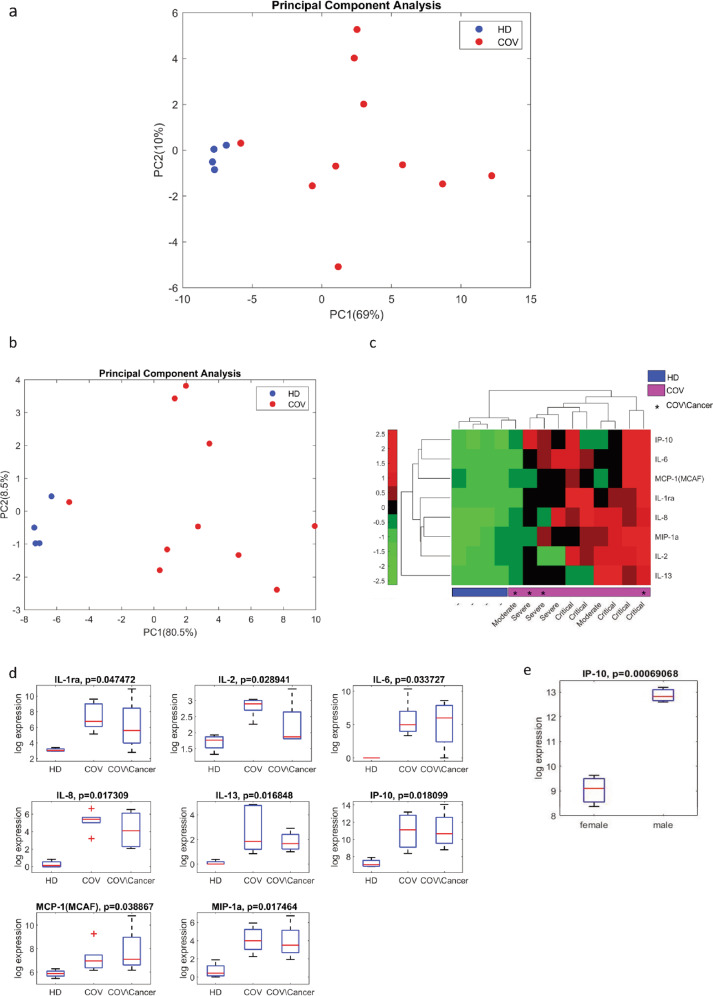
Cytokines multiplex analysis. **a** Principal component analysis based on a set of 27 cytokines separates HD (*n* = 4) from COV patients (*n* = 10) cohorts (IL-1β, IL-1ra, IL-2, IL-4, IL-5, IL-6, IL-7, IL-8, IL-9, IL-10, IL-12 (p70), IL-13, IL-15, IL-17, IP-10, MCP-1 (MCAF), MIP-1α, MIP-1β, PDGF-BB, RANTES, TNF-α, VEGF, FGF basic, Eotaxin, G-CSF, GM-CSF, IFN-γ). **b** Principal component analysis based on a set of eight cytokines which were found to be modulated between HD (*n* = 4) and COV patients (*n* = 10). **c** Unsupervised Hierarchical Clustering of the eight most modulated cytokines separates HD (*n* = 4) from COV patients (*n* = 10), COVID-19 disease severity, and cancer comorbidity are indicated in the graph. Clustering was built using Euclidean distance and average linkage on standardized expression. Higher values are shown in red, and lower expression levels are represented in green. **d** Box plot of the distributions of eight most modulated cytokines between HD (*n* = 4), COV (*n* = 6), and COV/cancer (*n* = 4) patients. **e** Box plot of the gender-dependent distributions of the IP-10 cytokine in COV patients (f = 3; m = 7).

Overproduction of pro-inflammatory cytokines occurs both in COVID-19 diseases and cancer [21, 22]. Herein, we aimed to assess whether COVID-19 modulates differently cytokine production in infected subjects with or without cancer. With the aid of Bio-Plex Pro Human Cytokine 27-Plex Immunoassay, we assessed concomitantly the expression of 27 cytokines in patient sera. As shown in Fig. 1a, principal component analysis (PCA) based on the quantification of the 27 cytokines clearly discriminated a cohort of mild-to-critical COVID-19 subjects from HD. By focusing on the top statistically ranked cytokines we evidenced a subset of 8 cytokines expressed at higher levels in the COVID-19 subjects than in HD sera (Fig. 1b–d). Interestingly, an increased upregulation of IP-10 could be observed in male COVID-19 subjects compared to the non-gender segregated group (Fig. 1e) while the same does not happen in HD (Supplementary Fig. S2). Next, we assessed the presence of these eight cytokines in sera from COVID-19 subjects concomitantly affected by cancer, and no statistically significant differences were evidenced between this group and COVID-19 subjects (Fig. 1d).

In aggregate, we documented that COVID-19 mild-to-severe patients exhibited cytokine overproduction typical of active viral infection and this occurs independently from the cancer comorbidity.

### Subclasses of hematopoietic cell populations are modulated in COVID-19 cancer patients

**Fig. 2:**
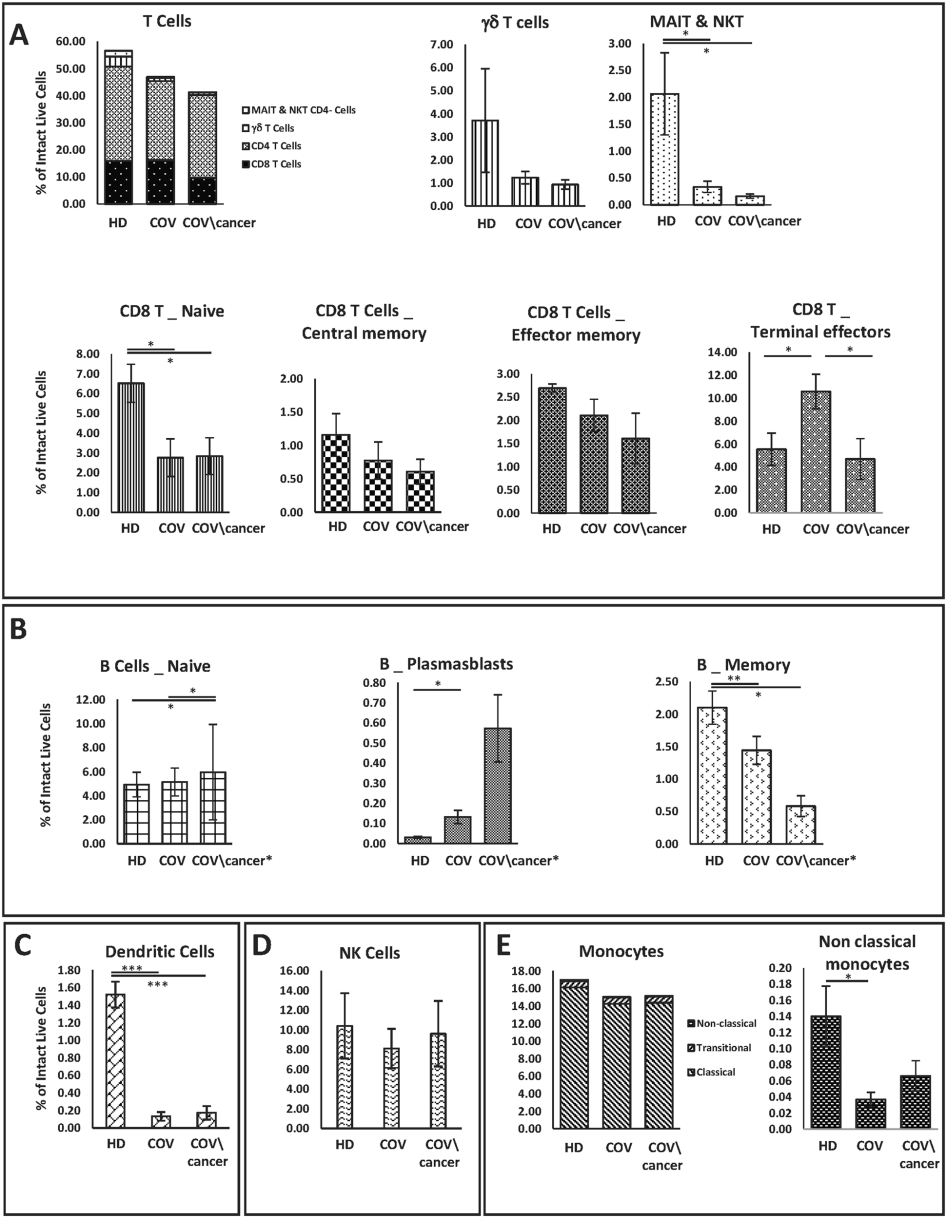
PBMCs phenotype by CyTOF. Intact live cell percentage of T (**A**), B (**B**)*, dendritic (**C**), NK (**D**) cell subpopulations, and monocytes (**E**). *Sand-006 patient with chronic lymphatic leukemia (CLL) is not included in (**B**). HD (*n* = 3) reveals a partially different populations and subpopulations phenotyping from disease-affected patients, both COV/cancer (*n* = 5) and COV (*n* = 6) cohorts. Data represent mean ± SEM. Student’s *t* test significance has been reported in graphs.

To characterize the immune phenotyping of COVID-19 patients, we carried out CyTOF analysis. The immune cellular subsets identified using a 30 antibody panel are listed in Supplementary Table II. T cells were reduced in COVID-19 patients compared to HD. This reduction was mainly due to a decrease of mucosal-associated invariant T cells (MAIT), NKT, and γδ T cells. The total amount of CD8^+^ T cells was almost unchanged, but an imbalance of cell subpopulations consisting of an increase in terminal effectors CD8 + T and a decrease of naive CD8^+^ T was evidenced (Fig. 2A). Among B cells, the minority subclass, plasmablasts, as expected robustly increased while B-memory cells slightly decreased in COVID-19 affected patients compared to HD (Fig. 2B). Within dendritic cells, myeloid and plasmacytoid subpopulations were clearly reduced in COVID-19 patients compared to HD (Fig. 2C). A slight reduction of NK cells (Fig. 2D) and non-classical monocytes (Fig. 2E) was observed in COVID-19 patients compared to HD. The majority of these modifications have been already reported in the literature confirming the reliability of our results [12, 23–29]. To search for specific hematopoietic alterations in COVID-19 patients with cancer, we analyzed their immune profiling and compared it to that of COVID-19 patients without cancer. A further slight reduction of T cells was observed in COVID-19 patients with cancer (Fig. 2A). This reduction was mainly due to terminal effectors CD8^+^ T cells that are back to the levels observed in HD, and MAIT-NKT cells. Regarding B cells, the imbalance observed in COVID-19 patients became more pronounced in cancer patients, being plasmablasts further increased and B-memory decreased (Fig. 2B). The total amount of dendritic (Fig. 2C) and NK (Fig. 2D) cells were comparable between COVID-19 patients with cancer and without cancer. The total amount of monocytes was almost unchanged, whereas the non-classical monocytes subpopulation was rather increased in COVID-19 cancer patients compared to non-cancer patients (Fig. 2E). A graphical representation of the CyTOF data in our cohorts is shown in the Cen-se plot, reporting that different cancer patients affected by different tumors exhibited peculiar differences in the quantity of the various immune cell subpopulations (Supplementary Fig. S3).

### Identification of a gene signature associated with COVID-19 patients with cancer

**Fig. 3:**
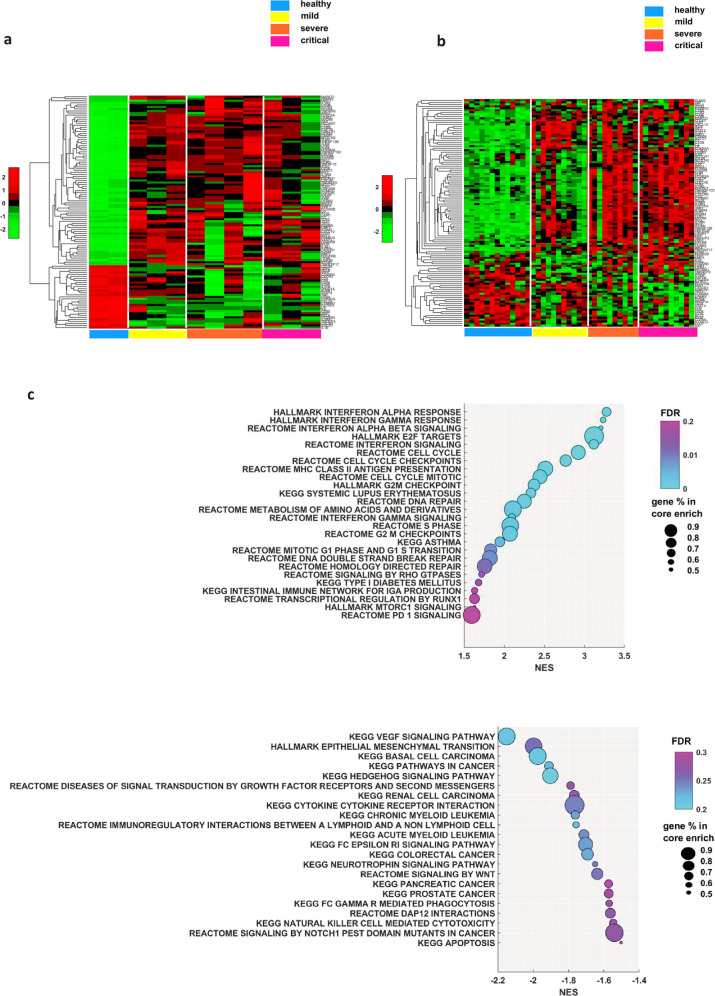
NanoString gene expression analysis. **a** Hierarchical clustering of 114 genes significantly modulated between HD (*n* = 2) and COV patients (*n* = 10) in Sant’Andrea Hospital cohort. Patients were sorted for the gravity of disease from healthy to critical status. HD reveals a different gene expression profile from disease-affected patients. **b** The same panel of genes was validated on a published cohort of 45 samples, including 13 HD and 32 COV patients. Hierarchical clustering showed a very similar expression profile. **c** Gene Set Enrichment Analysis (GSEA) was conducted considering a ranked list of all 750 genes of the NanoString PanCancer IO 360 Panel. Ranking was based on a score evaluated considering the sign of the modulation and the *P* value from permutation test between COV/cancer (*n* = 5) and COV patients (*n* = 5). Enriched gene set for activated genes and deactivated genes are represented on the left and on the right panel, respectively. Statistical significance is shown by a false discovery rate and represented as a color scale. Dimension of circles indicates the percentage of genes included in the gene set enrichment and all the pathways are sorted by the negative or positive normalized enrichment score (NES).

To further dissect the molecular features distinguishing COVID-19 patients with cancer from COVID-19 patients without cancer, we performed gene expression profiling of total RNA derived from PBMCs of both patient groups. We used the NanoString PanCancer IO 360 Panel that allows analyzing simultaneously the expression of 750 genes involved in the immune response. First, we identified 236 genes whose expression was modulated between COVID-19 patients (*n* = 10) and HD (*n* = 2). To further validate the robustness of our findings, we accessed the gene expression raw data of Terrier’s group who analyzed the immunological transcriptional signature of peripheral white blood cells of 50 COVID-19 patients with different degrees of severity [12]. Out of the 236 genes identified in our analysis, we could match 114 genes that overlap with those analyzed by Terrier’s group (Fig. 3a and Supplementary Table III). Notably, we found that the group of genes that differentiates COVID-19 patients (*n* = 50) from HD (*n* = 18) in the cohort reported in Hadjadj J. et al., 2020 is also able to discriminate COVID-19 subjects versus HD in our cohort (Fig. 3b). Of note, in the last analysis, our gene signature is clearly able to cluster COVID-19 patients on the basis of clinical severity, too.

Next, we aimed to identify genes whose expression was selectively modulated between COVID-19 patients with cancer or without cancer. Gene Set Enrichment Analysis (GSEA) carried out by looking at all ranked genes revealed surprisingly that genes belonging to interferon α, γ, and β pathways were upregulated in COVID-19 cancer patients (Fig. 3c, upper panel). Since it has been described that one hallmark of the SARS-CoV-2 infection is the ability of the virus to imbalance the IFN pathways [12, 15–17], this result surprisingly suggests that the concomitant presence of COVID-19 and cancer triggers a specific inflammatory response. Intriguingly, GSEA enriched for E2F cell cycle-related targets gene, cell cycle, G2M checkpoint, and DNA repair resulted to be upregulated in COVID-19 cancer patients when compared to those without cancer (Fig. 3c, upper panel).

**Fig. 4:**
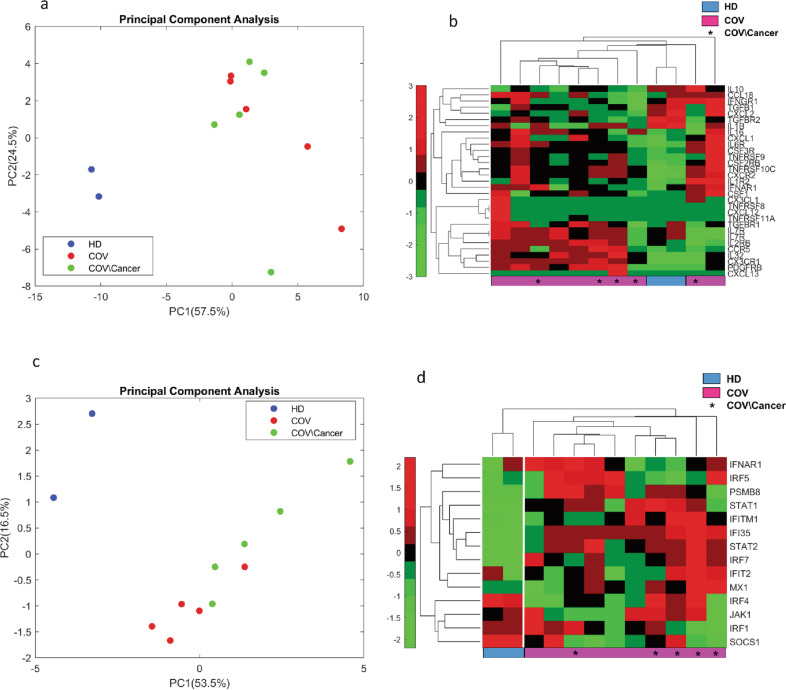
Evaluation of cytokines & chemokines and type I IFN-related genes signature on NanoString gene expression profile. **a**, **b** Principal component analysis and hierarchical clustering of a cytokines & chemokines gene signature reported in Hadjadj J. et al., 2020. The signature is able to discriminate HD (*n* = 2) from COV patients (*n* = 5). Cancer patients are also indicated (*n* = 5). **c**, **d** Principal component analysis and hierarchical clustering of a type I IFN gene signature reported in Hadjadj et al., 2020. The signature is able to discriminate HD (*n* = 2) from COV patients (*n* = 5). A difference is also evident between COV/cancer (*n* = 5) and COV patients (*n* = 5).

Hadjadj et al. reported two gene signatures, which were related to type I IFN-related genes and cytokine/chemokines, respectively, and allowed to classify COVID-19 symptomatic patients in three groups: mild-to-moderate, severe, and critical. By applying these signatures to our patient cohort, we found that they were able to discriminate COVID-19 patients from HD (Fig. 4a, b). Interestingly, unlike the cytokines/chemokines signature, type I IFN-related genes were modulated in COVID-19 cancer patients when compared to COVID-19 patients (Fig. 4c, d). This was evident in both principal component (PC)1 (53.5%) and PC2 (16.5%) analysis components (Fig. 4c).

**Fig. 5:**
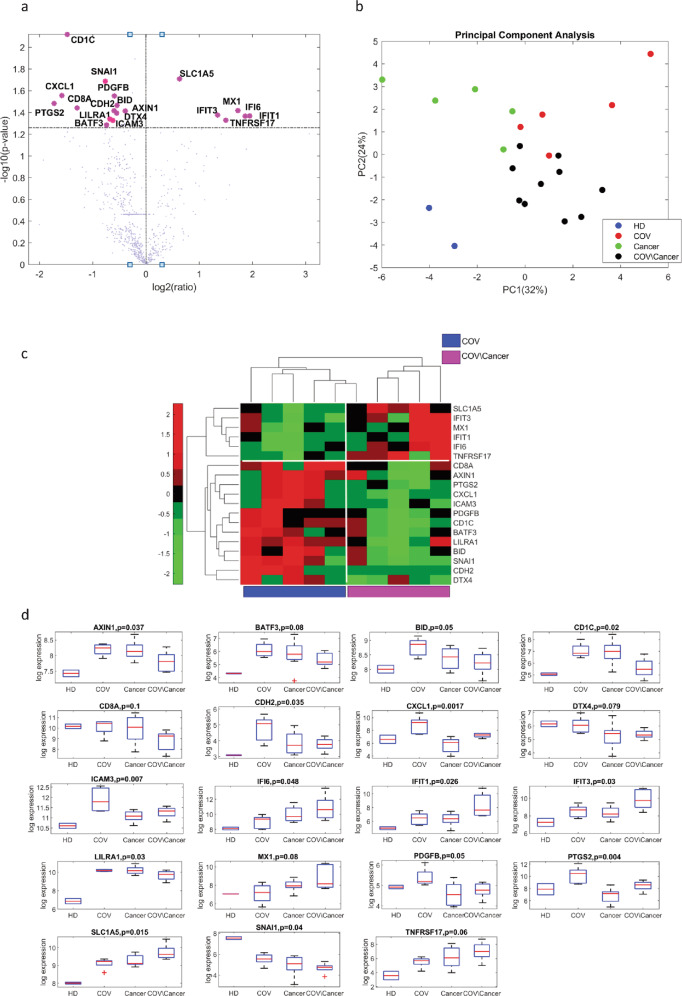
Gene expression signature modulated between COVID-19 patients affected by cancer and COVID-19 patients without cancer. **a** Volcano plot shows *P* values and related log2 fold change of genes comparing cancer patients and no-cancer patients, both affected by COVID-19. Statistically significance was evaluated by permutation test setting the threshold at 5%. Significant genes are highlighted. **b** Principal component analysis of the 19 genes on HDs (*n* = 2), COV (*n* = 5), cancer (*n* = 10), and COV/cancer (*n* = 5) patient groups. **c** Unsupervised Hierarchical Clustering of the 19-gene signature found to be significantly modulated between COV/cancer (*n* = 5) and COV patients (*n* = 5). The signature seems to be able to clearly separates the two groups of patients. **d** Box plot of the distributions of the 19-gene signature in HDs (*n* = 2), COV (*n* = 5), cancer (*n* = 10), and COV/cancer (*n* = 5) patient groups. Six genes were statistically significant upregulated:IFI6 (*P* = 0.042918), IFT1 (*P* = 0.042844), IFT3 (*P* = 0.042039), MX1 (*P* = 0.038236), SLC1A5 (*P* = 0.019546), and TNFRSF17 (*P* = 0.046937), while 13 were downregulated: AXIN1 (*P* = 0.038559), BATF3 (*P* = 0.051956), BID (*P* = 0.034231), CD1C (*P* = 0.0075958), CD8A (*P* = 0.036176), CDH2 (*P* = 0.038292), CXCL1 (*P* = 0.027772), LILRA1 (*P* = 0.040285), ICAM3 (*P* = 0.047029), DTX4 (*P* = 0.045546), PDGFB (*P* = 0.028081), PTGS2 (*P* = 0.032919), and SNAI1 (*P* = 0.020527).

Based on these findings, we looked for a gene expression signature capable to discriminate between COVID-19 patients with cancer or without cancer. Bioinformatics analysis allowed us to identify a signature composed of 19 genes whose expression was statistically significantly modulated in COVID-19 cancer patients (*n* = 5) with respect to both HD (*n* = 2) and COVID-19 subjects without cancer (*n* = 5) (Fig. 5a–d). Of note, the PCA analysis performed with COVID-19 cancer, COVID-19, and cancer patients clearly separates the three groups from each other and from HDs (Fig. 5b). A group of six genes was upregulated, whereas 13 were downregulated (Fig. 5a, c, d).

**Fig. 6:**
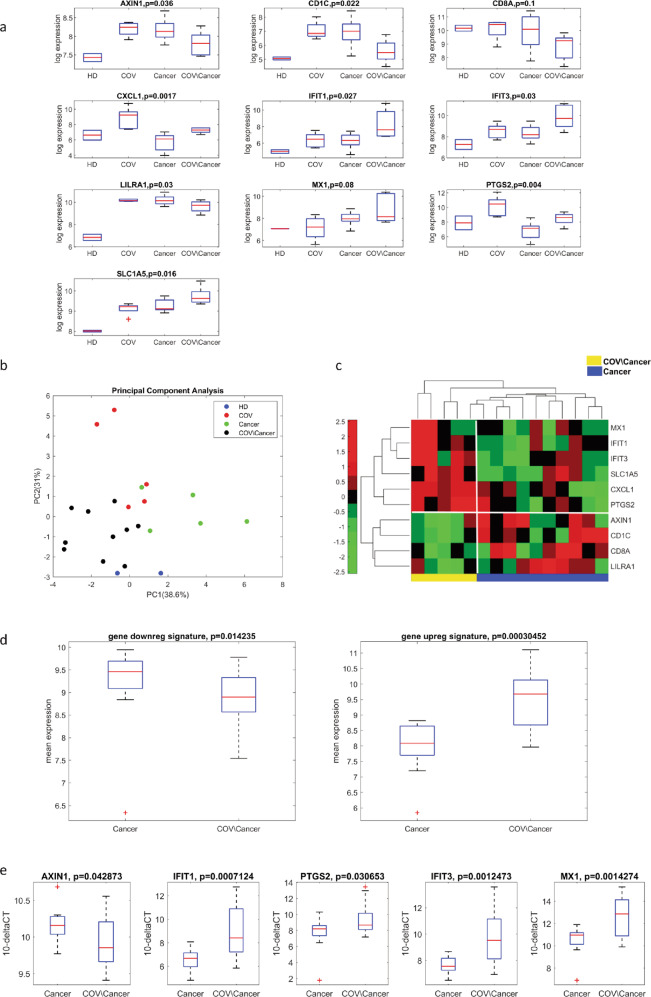
Evaluation of the gene signature on COVID-19 cancer patients. **a** Box plot of the distributions of 10 out 19-gene signature on HDs (*n* = 2), COV (*n* = 5), cancer (*n* = 10) and COV/cancer (*n* = 5) patients. **b** Principal component analysis of the 10 genes on HDs (*n* = 2), COV (*n* = 5), cancer (*n* = 10) and COV/cancer (*n* = 5) patient groups. **c** Unsupervised hierarchical clustering based on ten gene signature. The gene signature shows a different profile between cancer patients in presence or in absence of COVID-19 infection. **d** Box plot of the distributions of gene signature in cancer (*n* = 12) and COV/cancer (*n* = 21) validation cohorts. **e** Box plot of the distributions of AXIN1, IFIT1, PTGS2, IFIT3, MX1 in cancer (*n* = 12) and COV/cancer validation cohorts (*n* = 21).

To further validate the specificity of the genes significantly modulated in COVID-19 cancer patients, we carried out a similar analysis on the RNA from PBMCs of ten cancer patients negative for COVID-19. Notably, we found that 10 out of the 19 genes previously identified were also differentially regulated comparing COVID-19 cancer patients with cancer patients without COVID-19 (Fig. 6a–c). The majority of upregulated genes belong to the interferon signaling and cytotoxicity pathways while several downregulated genes fall into antigen presentation functional annotated category (https://www.nanostring.com/products/ncounter-assays-panels/oncology/pancancer-io-360/) (Supplementary Fig. S4)). To further validate the robustness of the gene signature obtained by Nanostring, we analyzed by real-time PCR the expressions of the ten genes of interest. In total, 18 new patients were included in the analysis with a final validation cohort consisting of 12 cancer and 21 COVID-19 cancer patients (Supplementary Fig. 1 and Supplementary Table I). Overall, the entire gene signature was confirmed, being downregulated and upregulated genes differentially expressed in the validation cohorts (Fig. 6d). Focusing on single genes, five out of ten genes (AXIN1, IFIT1, PTGS2, IFIT3, MX1) are significantly deregulated in the new COVID-19 cancer patient cohort (Fig. 6e). Interestingly, the validated gene signature is independent of cancer type comparing the gene signature in the lung cancer cohort (being the most representative) versus the other types of cancer (Supplementary Fig. S5). Intriguingly, two genes, PTGS2 and CXCL1, are upregulated in COVID-19 cancer patients in a gender-dependent-manner being upregulated in males but not in females (Supplementary Fig. S6).

In aggregate, our findings identify a restricted gene signature that was able to discriminate, in a confirmatory consecutive cohort, COVID-19 cancer patients from both COVID-19 patients without cancer and cancer patients without COVID-19.

**Fig. 7:**
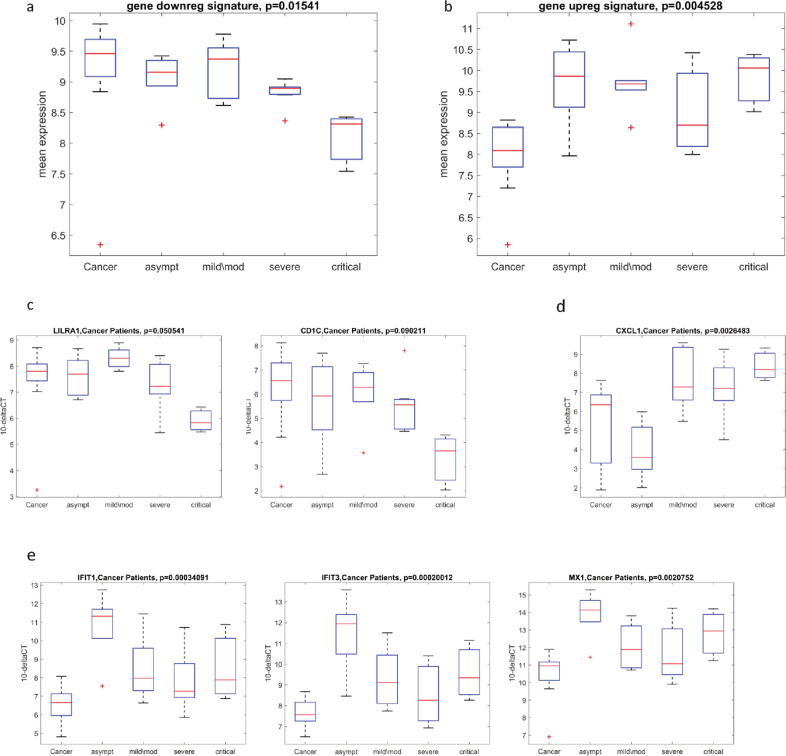
Evaluation of the gene signature on COVID-19 cancer patients in COVID-19 disease severity-manner. **a** Box plot of the COVID-19 disease severity-dependent distributions of downregulated gene signature in cancer (*n* = 12) and COV/cancer (*n* = 21) (asympt (*n* = 4), mild/mod (*n* = 7), severe (*n* = 7), critical (*n* = 3)) validation cohorts. **b** Box plot of the COVID-19 disease severity-dependent distributions of upregulated gene signature inn cancer (*n* = 12) and COV/cancer (*n* = 21) validation cohorts. **c** Box plot of the COVID-19 disease severity-dependent distributions of LILRA1 and CD1C in cancer (*n* = 12) and COV/cancer (*n* = 21) validation cohorts. **d** Box plot of the COVID-19 disease severity-dependent distributions of CXCL1 in cancer (*n* = 12) and COV/cancer (*n* = 21) validation cohorts. **e** Box plot of the COVID-19 disease severity-dependent distributions of IFIT1, IFIT3, and MX1 in cancer (*n* = 12) and COV/cancer (*n* = 21) validation cohorts.

Next, we subdivided COVID-19 cancer patients according to COVID-19 disease severity. Notably, the ten gene signature shows a severity-dependent trend for downregulated genes (Fig. 7a), while the same was not observed for upregulated genes (Fig. 7b). Focusing on single-gene expression, we observed that two genes LILRA1 and CD1C were significantly downregulated in a disease severity-dependent-manner (Fig. 7c). CXCL1 was downregulated in asymptomatic subjects while it was upregulated in mild/moderate, severe, and critical patients (Fig. 7d). IFIT1, IFIT3, and MX1 were upregulated in all severity degrees and further increased in asymptomatic subjects (Fig. 7e). These results indicate that a specific PBMC gene signature occurs in asymptomatic COVID-19 cancer patients, suggesting its usefulness in the identification of this status.

### An immune and proliferation transcriptional network is activated in COVID-19 cancer patients

**Fig. 8:**
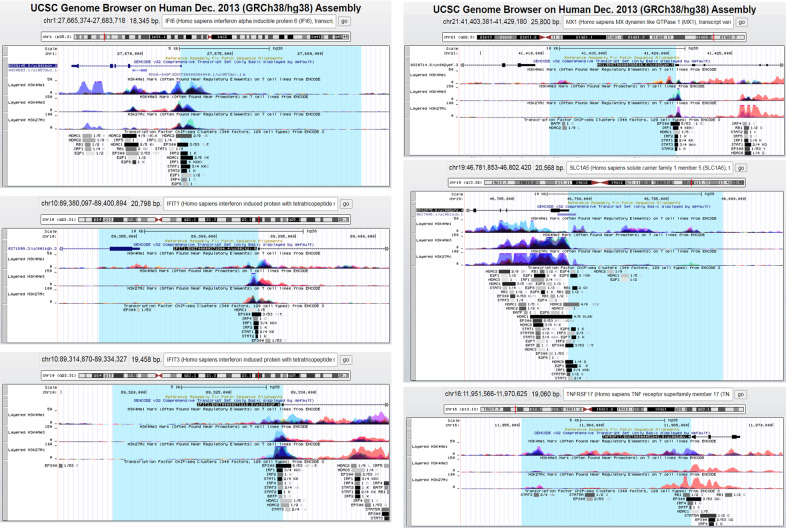
UCSC genome browser ChIP-seq analysis of the 6 upregulated promoters in COVID-19 cancer patients. Analysis on IFI6, IFIT1, IFIT3, MX1, SLC1A5, TNFRSF17 promoters using UCSC genome browser and selecting available cells of blood origin (GM12878 and K562 cell line). The analysis has been performed on a region spanning 10Kb upstream TSS (highlighted in light blue) and the first intron of the gene. The binding for the following transcriptional factors have been searched: BATF, E2F family, P300, GATA family, HDAC family, IRF family, RUNX family, STAT family, TEAD4, RB1. NFY, and SP1 ubiquitous factors were used as control. The H3K4me1, HeK4me3, and H3K27ac have been used to identify the euchromatin region around the start sites. The same analysis for the downregulated genes is shown in Supplementary Fig. S7.

Taking advantage of the Genome Browser ChIP-seq databases at the University of California Santa Cruz (UCSC) (ENCODE tracks), we looked for a common binding signature of transcription factors to the promoters of the genes modulated in COVID-19 cancer patients. The query to the UCSC genome browser has been restricted to the available cells of blood origin (Supplementary Table IV). Since in the GSEA analysis IFN α, γ, and β pathways as well as E2F cell cycle-related targets were the most significantly modulated, we focussed our attention on the IRF and E2F family members (Supplementary Table V). Up to nine IRF members have been identified [30]. We focused on IRF1–5 for which ChIP-seq data are available and found that IRF and E2F family members bind to 12 and 10 promoters out of 19, respectively. Of note, all upregulated genes in the COVID-19 cancer signature contain a binding site for IRF family members in the promoter region spanning 10.000 kb upstream the start site and the first intron (Fig. 8). IRF family members bind 5 out of 13 promoters of the downregulated genes (Table 1 and Supplementary Fig. S7). Based on the role of STAT family members on the interferon pathways [31], we also investigated their recruitment onto the 19 promoters. STATs form a family of 7 members while STAT ChIP-seq available data are for STAT1, 2, 3, and 5a (Supplementary Table V). STAT members are recruited on 14 out 19 promoter regions and, as in the case of IRF, all upregulated genes in the COVID-19 cancer signature contain a binding for STATs (Table 1 and Fig. 8). It has been recently shown that either cancer or SARS-CoV-2 infection mediates the activity of the IRF4-BATF pathway [32, 33] activating IRF-mediated transcription. In agreement, among the ten promoters bound by BATFs, eight are also bound by IRFs. On the contrary, the binding of E2F family members occurs on the regulatory regions of three upregulated and eight downregulated genes (Table 1, Fig. 8 and Supplementary Fig. S7). We also assessed the binding of acetylases (p300) and deacetylases (HDACs) proteins and observed both enzymes mostly bind the same promoter regions in agreement with the knowledge that the proper transcriptional activity of target promoters depends on the balanced activities of the two enzymes [34, 35] (Table 1, Fig. 8 and Supplementary Fig. S7). Reassuringly, the analysis of ChIP-seq datasets revealed in vivo direct binding of IRF, STAT, and E2F family members onto the indicated promoters. The concomitant binding of IRF and STAT on all promoters of the upregulated genes and five of the downregulated once indicates a transcriptional immune signature specifically acting on COVID-19 cancer patients. Moreover, the binding of promoters of both up- and downregulated genes suggests that IRFs, STATs, and E2Fs contribute to aberrant gene regulation in COVID-19 cancer patients either activation or repression of transcription. This effect has been already shown for other diseases [31, 36, 37].

**Table 1:** Binding of IRF, STATs, BATF, HDACs, p300 and E2Fs to the regulatory region of upregulated and downregulated genes specifically modulated in COVID-19 cancer patients compared to COVID-19 patients.

Upregulated genes in COVID-19 cancer patients compared to COVID-19 patients	IRF	STAT	BATF	HDAC	p300	E2F
IFI6	IRF1, IRF2, IRF4, IRF5	STAT1, STAT2	----	HDAC1, HDAC2	p300	E2F1, E2F6, RB1
IFIT1	IRF1, IRF2, IRF4	STAT1, STAT2	----	HDAC1	p300	----
IFIT3	IRF1, IRF2, IRF4	STAT1, STAT2, STAT3, STAT5a	BATF	HDAC1, HDAC2, HDAC6	p300	----
MX1	IRF1, IRF2	STAT1, STAT2, STAT3	BATF	HDAC1, HDAC2	p300	----
SLC1A5	IRF1, IRF2, IRF3	STAT1, STAT3, STAT5a	BATF	HDAC1, HDAC2, HDAC3, HDAC6	p300	RB1, E2F1, E2F4, E2F6, E2F7, E2F8
TNFRSF17	IRF4	STAT3, STAT5a	BATF	HDAC1	p300	RB1
**Downregulated genes in COVID-19 cancer patients compared to COVID-19 patients**	**IRF**	**STAT**	**BATF**	**HDAC**	**p300**	**E2F**
Axin1	IRF1	STAT5a	----	HDAC1, HDAC2	p300	E2F6, RB1
BATF3	IRF4	STAT3, STAT5a	BATF	HDAC1	p300	----
BID (3 kb)	----	STAT3	----	HDAC1, HDAC2		E2F6, RB1
CXCL1	----	STAT3	----	HDAC2	p300	E2F1, E2F6, RB1
PDGFb	----	----	BATF	HDAC1, HDAC2,		
CDH2			BATF	HDAC1, HDAC2	p300	----
CD1C	IRF4	STAT3, STAT5a	BATF	----	p300	----
ICAM3	----	----	----	HDAC1,2	P300	E2F1
PTGS2	----	STAT3	----	----	p300	----
CD8a	IRF3, IRF4	STAT1	BATF	HDAC1, HDAC2	----	E2F6, RB1
DTX4	IRF1, IRF3, IRF4, IRF5	STAT1, STAT3, STAT5a	BATF	HDAC1, HDAC2	p300	E2F1,E2F6, E2F8, RB1
LILRA1	----	----	----	----	----	----

In aggregate, we identify a transcriptional network coupling innate immune response and cell proliferation in COVID-19 cancer patients.

## Discussion

In the present manuscript, we aimed to provide initial insights on the impact of COVID-19 on immune-circulating cells in cancer patients. Unexpectedly we unveiled a common gene signature in patients with different neoplasms and different degrees of COVID-19 disease. Albeit limited in number (COVID-19 *n* = 12 and COVID/Cancer *n* = 21), our COVID/Cancer cohort is to date the largest described where gene expression has been investigated in depth. We are aware that the heterogeneity of our COVID/Cancer cohort may result in the loss of cancer-specific gene signatures, however, we believe that the finding of a gene expression signature common to different cancer types and diseases stages could reveal interesting aspects worth further investigation.

To date, there are still scarce evidence on the molecular basis of the severe adverse effects to which COVID-19 cancer patients undergo. Uncertain results emerge from the comparison of both ACE2 and TMPRSS2 expression and functional activities between COVID-19-positive subjects and cancer patients [38, 39]. The aberrant immune response is a common event for both SARS-CoV-2 infection and cancer. This prompted an unprecedented research focus to identify immune factors that discriminate COVID-19 patients with or without cancer. Herein, we observed that serum production of well-recognized inflammatory mediators of the “hypercytokinemia” typical of COVID-19-positive subjects [40] were not differentially affected by the cancer status of SARS-CoV-2-infected patients. CyTOF cell subpopulation immune-profile revealed a small reduction of T cells in COVID-19 patients, further evidenced in COVID-19 cancer patients. This reduction was mainly due to terminal effectors CD8^+^ T cells whose amount in COVID-19 cancer patients became similar to that of HD, and MAIT-NKT cells [41, 42]. A strong reduction of T cells has been described in severe/critical COVID-19 patients [43], while we observe a small reduction of these cells. One explanation should reside in the clinical severity heterogeneity in our COVID-19 cohort. Regarding B cells, the imbalance observed in COVID-19 patients became worse in COVID-19 cancer patients, being plasmablasts further increased and B memory decreased.

Notably, we identified a five-gene signature that was modulated between COVID-19, cancer, and COVID-19 cancer patients. From a functional point of view, genes involved in interferon signaling and cytotoxicity (IFIT1, IFIT3, and MX1) are upregulated in COVID/cancer cohort. On the other end, genes involved in antigen presentation are downregulated, indicating that in patients with both diseases the inhibition of the immune response is even more pronounced than in patients with only one of the two, suggesting a synergy of action of the two diseases.

GSEA analysis highlighted that the most significantly upregulated pathways between COVID-19 and COVID-19 cancer patients are those induced by interferon. In agreement with the fact that upregulated gene signature in COVID-19 cancer patients is enriched for interferon-regulated genes, their promoters are bound by key regulators of interferon gene expression, IRF, STAT, and BATF TFs. This further supports that. IFN pathways are deregulated in COVID-19 patients [17, 44]. On the other side, IFN pathways are usually active in cancer [45, 46]. Their further induction in COVID-19 cancer patients could indicate a functional synergy between COVID-19 and cancer suggesting that the active IFN pathways in these patients may be one of the factors influencing the increased disease severity observed in COVID\cancer patients.

Another pathway significantly upregulated in the GSEA analysis is that of E2F cell cycle-related targets. E2F family members are key regulators of proliferation and several promoters of upregulated genes in COVID-19 cancer patients are bound by one or more of these proteins. An in vitro phospho-proteomic approach on cells infected with SARS-CoV-2 indicates that cell cycle kinases are downregulated, suggesting a general mitotic arrest [47]. On the contrary, the aberrant regulation of the cell cycle is a hallmark of cancer [48]. We can speculate that the concomitant binding of TFs regulating the inflammatory response and TFs regulating proliferation on many of the gene promoters modulated in COVID-19 cancer patients may lead to a wide dramatic rearrangement of different signaling pathways.

The analysis of the TFs in vivo binding on the promoters of the gene signature suffers of limitations. First, the data deposited in the database are referring to cells different from the PBMCs of our patient cohort. Furthermore, there are no ChIP-seq experiments for all members of the TF families we analyzed. For example, there are data only for IRF1 to 5 and STAT1–3 and 5a. However, we consider that the bindings we have identified are not artifacts. We have indeed also looked for other TFs related to inflammation [44], proliferation (NFY) [49], and ubiquitously expressed (Sp1) [50] (Supplementary Table IV) and none of them bind the promoters of interest.

The introduction of vaccination is rapidly progressing and cancer patients are among the fragile subjects who have been prioritized to receive it. We are already seeing a reduction in severe COVID-19 patients and therefore also in cancer patients. However, it is becoming more and more clear that not all cancer patients are responding properly to vaccination. In particular, onco-hematologic patient cohorts, undergoing different treatments, do not respond adequately [51, 52]. In the light of these evidence, it is still of great importance to study the molecular mechanisms and to collect information on the interaction between the SARS-CoV-2 virus and cancer diseases.

## Materials and methods

### Multiplex cytokine immunoassay

The Bio-Plex Pro Human Cytokine 27-Plex Immunoassay (Bio-Rad Laboratories S.r.l. Segrate, Milan, Italy) was used to characterize Cytokine Immunoassay of human sera samples. Samples were prepared following the manufacturer’s instructions. The following Cytokine were analyzed: FGF basic, Eotaxin, G-CSF, GM-CSF, IFN-γ, IL-1β, IL-1ra, IL-2, IL-4, IL-5, IL-6, IL-7, IL-8, IL-9, IL-10, IL-12 (p70), IL-13, IL-15, IL-17, IP-10, MCP-1 (MCAF), MIP-1α, MIP-1β, PDGF-BB, RANTES, TNF-α, VEGF. Sample acquisition was performed using the Bio-plex Manager MP software with the MagPix instrument. Data were analyzed with xPONENT software.

### Isolation of peripheral blood mononuclear cells (PBMCs)

Isolation of Human PBMCs was done from peripheral blood, supplemented with anticoagulants (EDTA), obtained from both COVID-19 patients and HD. All patients voluntarily agreed to participate to the study and signed an informed consent form. The detailed procedure is described in the Supplementary informations.

### Immune phenotyping by CyTOF and data analysis

Maxpar Direct Immune Profiling Assay was used to characterize immune phenotyping of PBMC samples. Samples were prepared following the manufacturer’s instructions. Sample acquisition was performed using the Helios system. A list of used antibodies is presented in Supplementary informations.

Data were analyzed by MaxparR Pathsetterrh (Fluidigm, South San Francisco, CA, USA), powered by Gem Stone™ 2.0.41 (Verity Software House, Topsham, ME, USA), and normalized using the CyTOF Software v.6.7.1016. The high-dimensional Cen- se′™ (next-gen t-SNE) map has been used to identify and visualize all populations of immune cells in our samples.

### Gene expression analysis

Total RNA was extracted from blood samples, using Qiazol (Qiagen, Hilden, Germany), purified from DNA contamination through a DNase I (Qiagen) digestion step, and further enriched by Qiagen RNeasy columns profiling (Qiagen). The quantity and purity of the RNA were assessed with the NanoDrop spectrophotometer. In the case of low 260/230 ratios, the samples were re-purified by chloroform and subsequent ethanol precipitation. The quality of the RNA was controlled with the Bioanalyzer employing the Agilent RNA 6000 Pico or Nano Kit (Agilent Technologies, Santa Clara, CA, USA). Gene expression was measured using the NanoString PanCancer IO 360 Panel. As input 30–100 ng total RNA was used following the manufacturer’s instructions. After the Codeset hybridization, the samples were washed and loaded on the cartridge within the Prep Station and subsequently analyzed with the nCounter Digital Analyzer.

### Bioinformatics analysis

RCC files were analyzed using nSolver analysis software (Version 4.0) as the manufacturer’s protocols. Negative and positive controls included in probe sets were used for background thresholding and a geometric mean of internal reference genes was used for normalization. Normalized counts were further analyzed by MATLAB R2019b. Hierarchical clustering by Euclidean distance and average linkage and principal component analysis were performed on *z* score transformed counts. Differentially expressed genes were detected using a permutation test and confirmed by a Wilcoxon rank-sum test. Kruskal–Wallis test was applied to evaluate differences among more than groups. A false discovery rate procedure was applied for multiple comparisons.

### Pathway analysis

A Gene Set Enrichment Analysis (GSEA software; https://www.gsea-msigdb.org/gsea/index.jsp) was conducted by using the curated gene sets of the Molecular Signature Database (MSigDB) derivated from KEGG, Hallmark, Reactome, and Biocarta collections. GSEA was run in preranked mode using classic as metric and 1000 permutations.

CHIP data were consulted from Transcription Factor ChIP-seq Clusters ENCODE 3 database (Source data version: ENCODE Nov 3, 2018) in UCSC Genome Browser (on Human Dec. 2013 (GRCh38/hg38) Assembly). A detailed description of the used strategy is reported in Supplementary informations.

### RNA extraction, cDNA synthesis, and RT-qPCR

Total RNA from PBMC samples was extracted using the Qiazol Lysis Reagent (Qiazol) and miRNeasy Mini Kit (Qiazol) following the manufacturer’s instructions. The first-strand cDNA was synthesized according to the instructions for the M-MLV RT kit (Invitrogen). Real-time quantitative PCR (RT-qPCR) was performed using TaqMan Fast Advanced Master mix (Applied Biosystems) on an ABI Prism 7900 apparatus (Applied Biosystems). Following TaqMan Gene Expression Assay (FAM)(ThermoFisher) were used: AXIN1 (Hs00394718_m1); CD1C (Hs00233509_m1); CD8A (Hs00233520_m1); CXCL1 (Hs00236937_m1);IFIT1 (Hs00356631_g1); IFIT3 (Hs00155468_m1); LILRA1 (Hs04401156_gH); MX1 (Hs00182073_m1); PTGS2 (Hs00153133_m1); SLC1A5 (Hs00194540_m1); PUM1 (Hs00472881_m1); SDHA (Hs00188166_m1). mRNA expression was normalized for PUM1 and SDHA geometric means. Relative mRNA expression was calculated using the comparative Ct method (10-deltaCT).

## Supplementary information

Supplementary informations

## Data Availability

The authors declare that all data supporting the findings of this study are available within the paper and its Supplementary informations files. NanoString data are available at Gene Expression Omnibus database with access ID GSE164571.
