# Supplementary material for: Multi-omic approach identifies a transcriptional network coupling innate immune response to proliferation in the blood of COVID-19 cancer patients
Source: Cell Death Dis. 2021 Oct 29;12(11):1019. doi: 10.1038/s41419-021-04299-y (PMC8553595; doi:10.1038/s41419-021-04299-y)
Supplement: Supplementary file 1 — Supplementary informations [file 41419_2021_4299_MOESM1_ESM.docx]

**Multi-omic approach identifies a transcriptional network coupling innate immune response to proliferation in the blood of COVID-19 cancer patients.**

Andrea Sacconi^1,13^, Claudia De Vitis^2,13^, Luisa de Latouliere^3,13^, Simona Di Martino^4^, Francesca De Nicola^3^, Frauke Goeman^3^, Carla Mottini^3^, Francesca Paolini^5^, Michela D’Ascanio^2^, Alberto Ricci^2^, Agostino Tafuri^2^, Paolo Marchetti^2^, Arianna Di Napoli^2^, Luciano De Biase^2^, Andrea Negro^2^, Christian Napoli^6^, Paolo Anibaldi^7^, Valentina Salvati^8^, Darragh Duffy^9^, Benjamin Terrier^10^, Maurizio Fanciulli^3^, Carlo Capalbo^2^, Salvatore Sciacchitano^2,11^, Giovanni Blandino^12^, Giulia Piaggio^3*^, Rita Mancini^2,14^ and Gennaro Ciliberto^8,14^.

^1^ UOSD Clinical Trial Center, Biostatistics and Bioinformatics, Regina Elena National Cancer Institute - IRCCS – Rome

^2^ Department of Clinical and Molecular Medicine, Sant'Andrea Hospital, Sapienza University of Rome, Rome, Italy.

^3^ UOSD SAFU, IRCCS - Regina Elena National Cancer Institute; Via Elio Chianesi 53, 00144, Rome, Italy.

^4^ Department of Pathology, IRCCS Regina Elena National Cancer Institute, Rome, Italy.

^5^ UOSD Tumor Immunology and Immunotherapy, IRCCS Regina Elena National Cancer Institute, Via Elio Chianesi 53, 00144, Rome, Italy.

^6^ Department of Medical-Surgical Sciences and of Translational Medicine, Sapienza University of Rome, Sant’Andrea Hospital, Rome, Italy

^7^ Hospital Direction and Clinical Departments, Sant’Andrea University Hospital, Rome, Italy

^8^ Scientific Direction, IRCCS Regina Elena National Cancer Institute, Rome, Italy.

^9^ Institut Pasteur, Laboratory of Dendritic Cell Immunobiology, Department of Immunology, Paris, France.

^10^ Department of Internal Medicine, National Referral Center for Rare Systemic Autoimmune Diseases, Assistance Publique Hôpitaux de Paris-Centre, University of Paris, Paris, France.

^11^ Laboratory of Biomedical Research, Niccolò Cusano University Foundation, Rome, Italy

^12^ UOSD Oncogenomica ed Epigenetica, IRCCS - Regina Elena National Cancer Institute; Via Elio Chianesi 53, 00144, Rome, Italy

^13^ These authors contributed equally

^14^ These authors contributed equally

* corresponding author [giulia.piaggio@ifo.gov.it](mailto:giulia.piaggio@ifo.gov.it)

**SUPPLEMENTARY MATERIAL AND METHODS**

**Isolation of Peripheral blood mononuclear cells (PBMCs)**

PBMC were isolated by density gradient centrifugation, using Lympholyte®-H Cell Separation Media (Cedarline). The blood samples were diluted with 1:2 volumes of phosphate-buffered saline (PBS), and gently layer the whole blood over top of the Lymphocyte (density gradient medium with ρ = 1.077 g/ml) making sure not to mix the two layers, before centrifugation at 1600 g for 20 minutes at 4°C in a swinging bucket rotor without brake. Most of the upper layer was then aspirated, leaving the mononuclear cells at the interphase, the mononuclear cells were transferred to a new 50 ml tube filled with PBS, and centrifuged at × 1600 g for 10 min at 4 °C (twice). When most of the platelets were removed, the PBMC were stored in two different storage media. Prior to storage, the batches were aliquoted in numbers and volumes that were appropriate for each protocol. A suspension of 107 PBMCs/ml was preserved in freezer medium, and a final concentration of 2x106 PBMCs/ml was suspended in Qiazol.

**Immune Phenotyping by CyTOF and data analysis**

Following antibodies were analyzed: CD45, CD196/CCR6, CD123, CD19, CD4, CD8a, CD11c, CD16, CD45RO, CD45RA, CD161, CD194/CCR4, CD25, CD27, CD57, CD183/CXCR3, CD185/CXCR5, CD28, CD38, CD56/NCAM, TCRgd, CD294, CD197/CCR7, CD14, CD3, CD20, CD66b, HLA-DR, IgD, CD127, Live/dead intercalator-103Rh.

**Pathway analysis**

Analyses were carried out on GM12878 cell line (ATCC® NIST-8398™) of B-Lymphocytes EPV-transformed and in K562 cell line (ATCC® CCL-243™) chronic myelogenous leukaemia (CML) bone marrow lymphoblast for the following transcriptional factors: BATF, E2F1, E2F4, E2F6, E2F7, E2F8, EP300, GATA1, GATA2, GATAD2A, GATAD2B, HDAC1, HDAC2, HDAC3, HDAC6, IRF1, IRF2, IRF3, IRF4, IRF5, NFYA, NFYB, RUNX1, RUNX3, STAT1, STAT2, STAT3, STAT5A, TEAD4, RB1, SP1. Transcription factors were checked within the first intron and 10kb upstream of the first exon, excepted for BID for which we analyzed 3kb.

TNF-a

**Supplementary Figure S1**

**4 HD**

**21 Cancer/COV**

**6 COV**

**12 Cancer**

**43 subjects**

**COVID-19 severity degree:**

**4 Asympt**

**9 Mild/mod**

**8 Severe**

**6 Critical**

**Cancer types:**

**13 Lung cancer**

**6 Hematologic malignancy**

**5 Gastrointestinal cancer**

**2 Breast cancer**

**2 CCRCC carcinoma**

**5 Other malignancies**

Schematic representation of analyzed cohorts. HD stands for Haelthy Donors, COV stands for COVID-19 patients, CCRCC carcinoma stands for Clear Cell Renal Cell Carcinoma.

**Supplementary Figure S2**

**
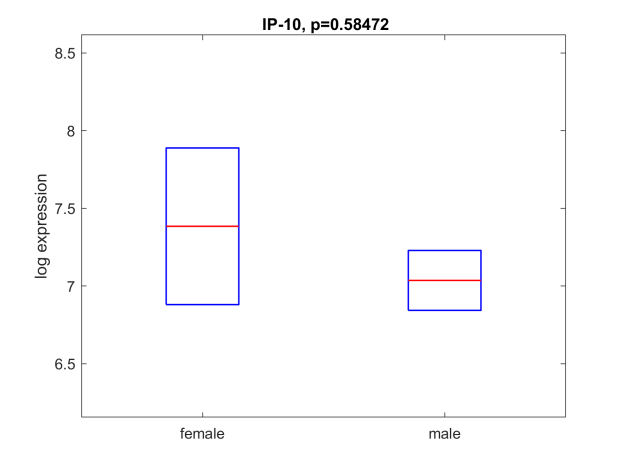
**

Box plot of the gender-dependent distributions of the IP10 cytokine in HD.

**Supplementary Figure S3**

Cen-se plot of the CyTOF data of HD, COV\cancer and COV patients. Cancer patients affected by different tumors exhibit peculiar differences in the quantity of the different immune cell subpopulations.

**Supplementary Figure S4**

**COVID/Cancer versus COVID**

**FUNCTIONAL ANNOTATION**

**Interferon signal (4/6) IFI6, IFIT1, IFIT3, MX1**

**Cytotoxicity (4/6) IFI6, IFIT1, IFIT3, MX1**

**Antigen presentation (4/13) BATF3 CD1C CD8A CXCL1**

**COVID/Cancer versus cancer**

**FUNCTIONAL ANNOTATION**

**Interferon signal (3/5) IFIT1, IFIT3, MX1**

**Cytotoxicity (3/5) IFIT1, IFIT3, MX1**

**Antigen presentation (2/4) CD1C CD8A**

The majority of upregulated genes belong to the interferon signaling and cytotoxicity pathways while several downregulated genes fall into antigen presentation functional annotated category. Upregulated genes are written in red, downregulated genes are written in blue

**Supplementary Figure S5**


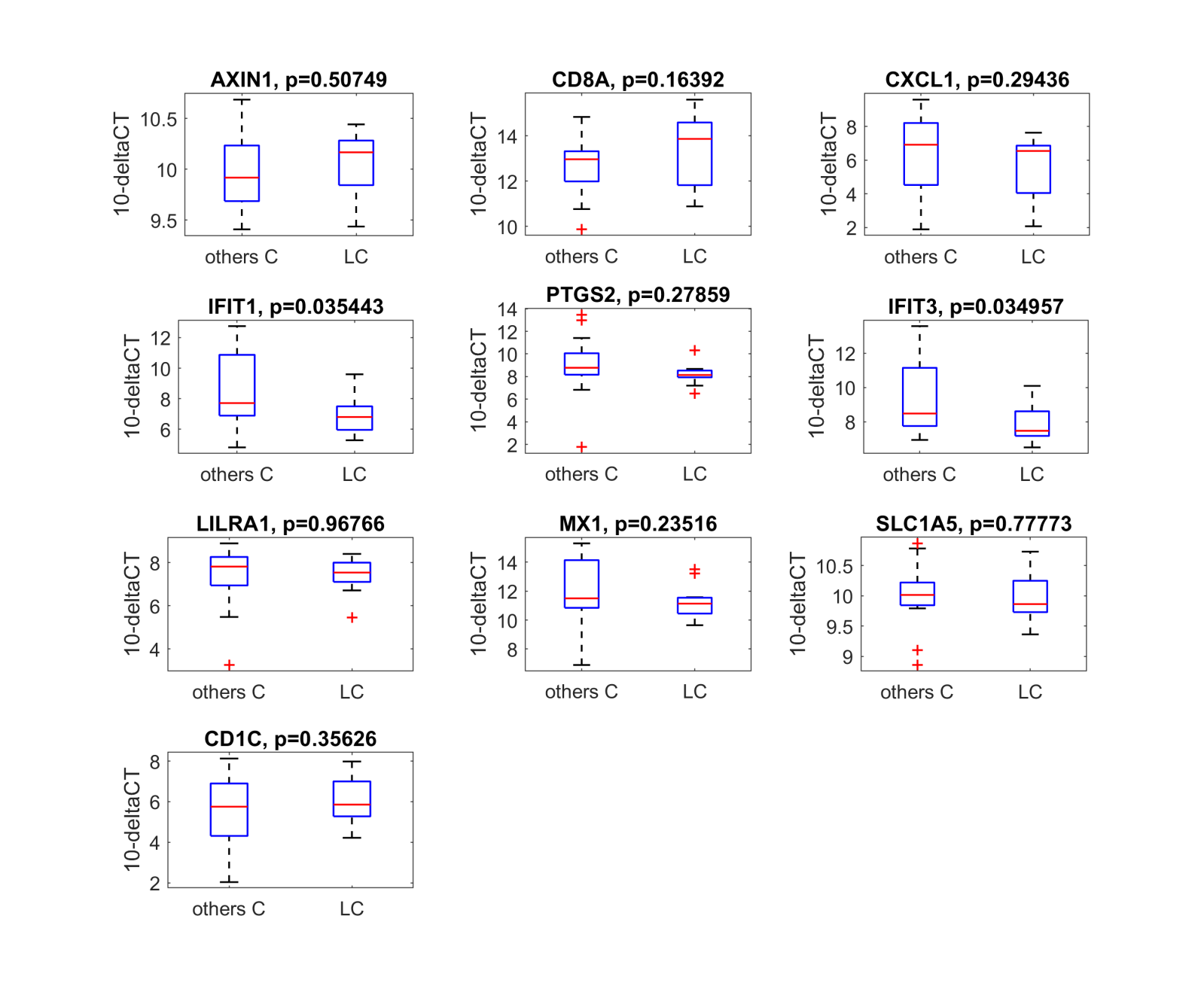


Box plot of the distributions of the gene signature in the lung cancer cohort versus the other type of cancer.

**Supplementary Figure S6**


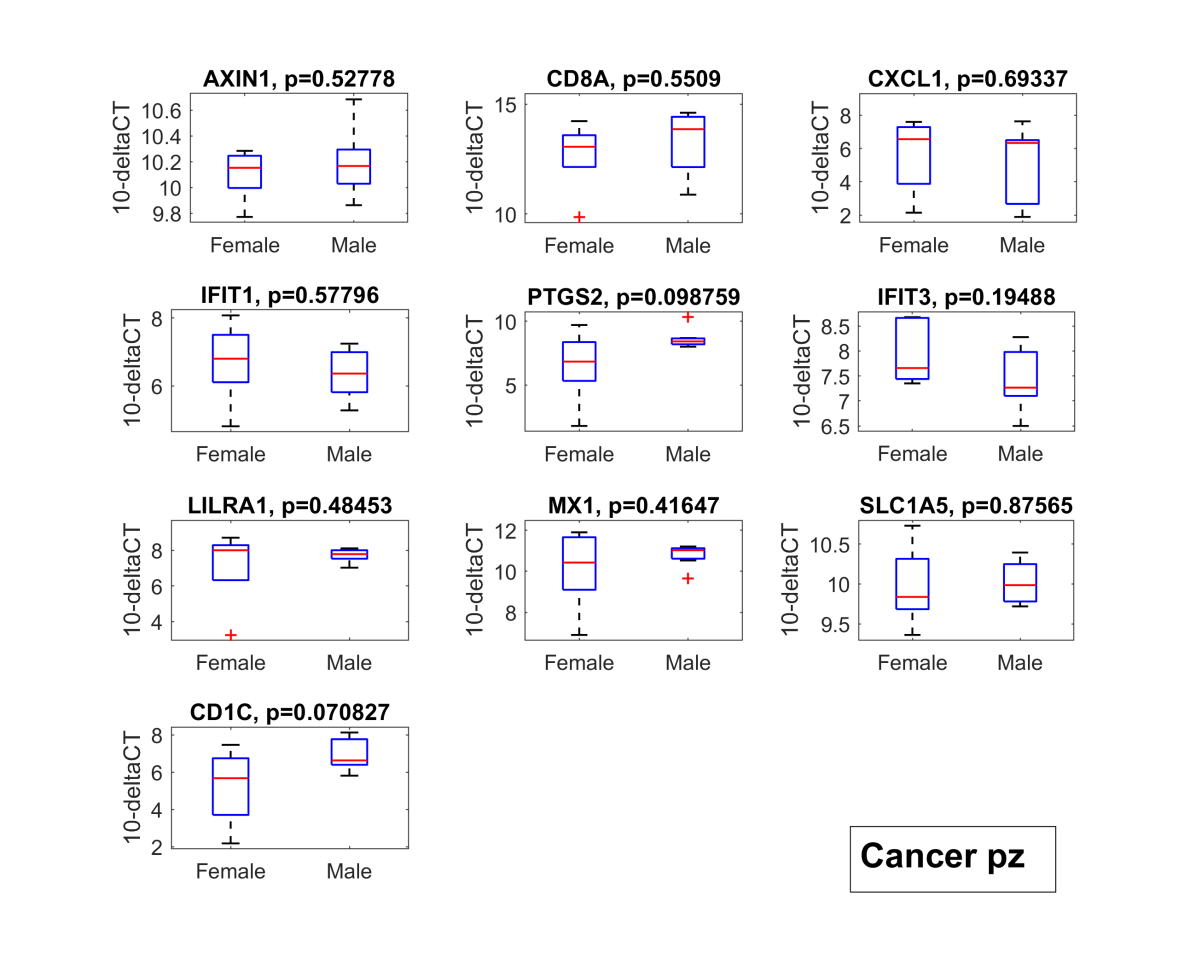

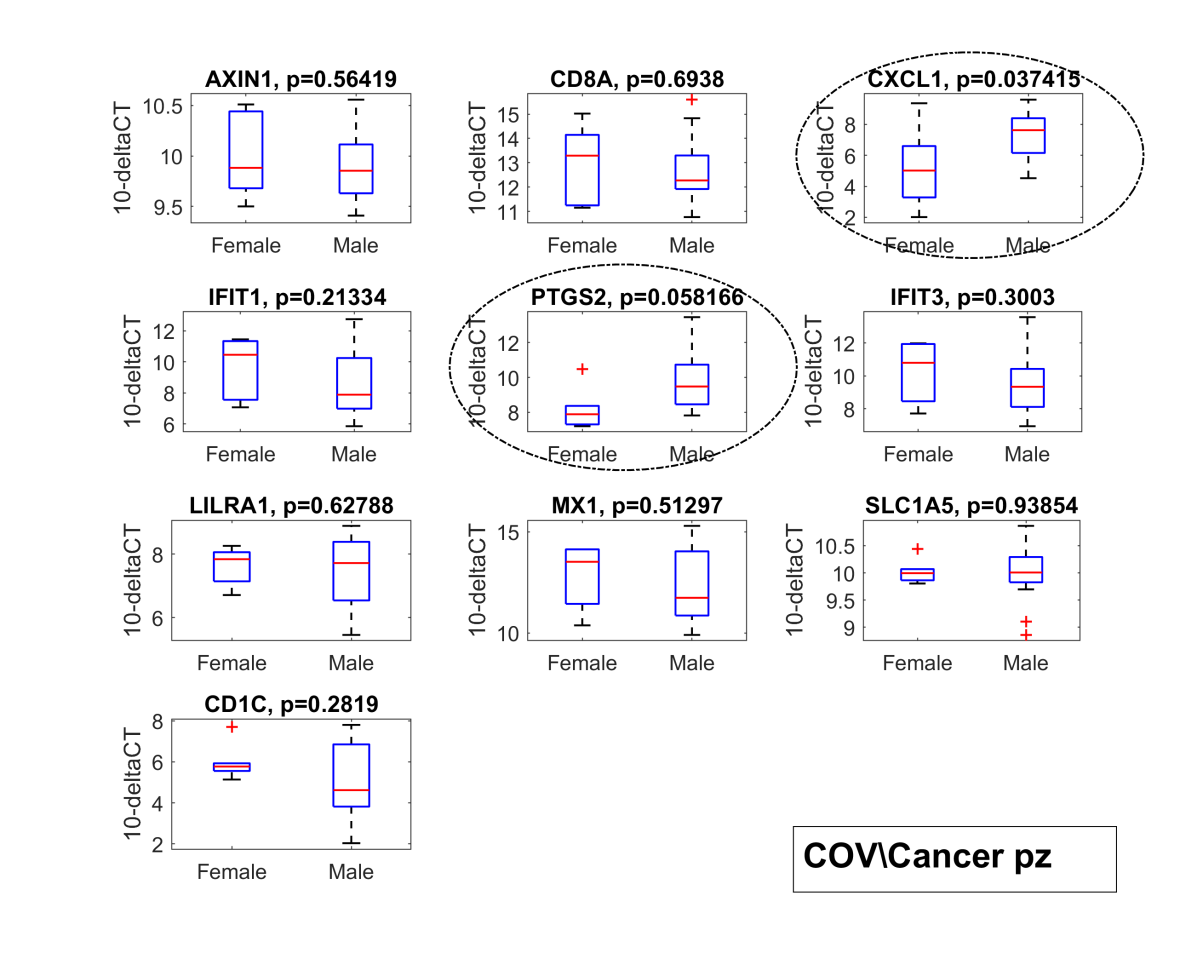


Box plot of the gender-dependent distributions of the gene signature in cancer and in COV\cancer groups. PTGS2 and CXCL1 genes (red circled) that are upregulated in COVID-19 cancer patients, are further upregulated in males than in females.

**Supplementary Figure S7**

UCSC genome browser ChIP-seq analysis of downregulated genes in COV\cancer versus COV patients. The analysis has been performed on region spanning 10Kb upstream TSS, highlited in light blue and the first intron of the gene.

**Supplementary Table I**

| Group | Pts | Sex | Age | Neoplastic Disease | Degree of Severity | Analysis | Anticancer treatment | Days between COVID-19 first positive swap and blood collection |
| --- | --- | --- | --- | --- | --- | --- | --- | --- |
| Healthy Donors  (53 y mean  age) | **HD 2** | **M** | **51** | **N.A.** | **N.A.** | **Cytof**  **Multiplex** | **N.A.** | **N.A.** |
|  | **HD Y** | **F** | **54** | **N.A.** | **N.A.** | **Cytof Multiplex Nanostring** | **N.A.** | **N.A.** |
|  | **HD 7** | **M** | **51** | **N.A.** | **N.A.** | **Cytof**  **Multiplex Nanostring** | **N.A.** | **N.A.** |
|  | **HD W** | **F** | **54** | **N.A.** | **N.A.** | **Multiplex** | **N.A.** | **N.A.** |
| Covid-19  (74,2 y  mean  age) | **Sand-010** | **M** | **65** | **N.A.** | **Mild** | **Cytof**  **Multiplex**  **Nanostring** | **N.A.** | **2** |
|  | **Sand-007** | **F** | **88** | **N.A.** | **Moderate** | **Cytof**  **Multiplex**  **Nanostring** | **N.A.** | **37** |
|  | **Sand-100** | **M** | **68** | **N.A.** | **Severe** | **Cytof**  **Multiplex**  **Nanostring** | **N.A.** | **39** |
|  | **Sand-002** | **M** | **95** | **N.A.** | **Critical** | **Cytof**  **Multiplex** | **N.A.** | **62** |
|  | **Sand-003** | **M** | **60** | **N.A.** | **Critical** | **Cytof**  **Multiplex**  **Nanostring** | **N.A.** | **37** |
|  | **Sand-004** | **F** | **69** | **N.A.** | **Critical** | **Cytof**  **Multiplex**  **Nanostring** | **N.A.** | **57** |
| Covid-19  Cancer  (66,5 y mean  age) | **IRE-COV 5571** | **F** | **34** | **Breast cancer** | **Asymptomatic** | **Rt PCR** | **No treatment**  **(neo-diagnosis)** | **4** |
|  | **IRE-COV 5572** | **M** | **67** | **Melanoma** | **Asymptomatic** | **Rt PCR** | **No treatment**  **(neo-diagnosis)** | **4** |
|  | **IRE-COV 5693** | **F** | **86** | **Breast cancer** | **Asymptomatic** | **Rt PCR** | **Cisplatinum plus gentamicine before Covid** | **4** |
|  | **IRE-COV 5694** | **M** | **62** | **Lung cancer - Bladder cancer** | **Asymptomatic** | **Rt PCR** | **No treatment**  **(neo-diagnosis)** | **4** |
|  | **Sand-009** | **F** | **74** | **Gastrointestinal Cancer** | **Mild** | **Cytof**  **Nanostring**  **Rt PCR** | **No treatment**  **(neo-diagnosis)** | **2** |
|  | **SA-COV30** | **M** | **44** | **Gastrointestinal Cancer** | **Mild** | **Rt PCR** | **Radiotherapy** | **27** |
|  | **SA-COV31** | **M** | **79** | **Bladder Cancer** | **Mild** | **Rt PCR** | **No treatment**  **(TURB)** | **21** |
|  | **SA-COV34** | **M** | **55** | **Gastrointestinal Cancer** | **Mild** | **Rt PCR** | **No treatment** | **23** |
|  | **SA-COV35** | **F** | **69** | **Lung Cancer** | **Mild** | **Rt PCR** | **Radiotherapy** | **10** |
|  | **SA-COV33** | **M** | **52** | **Gastrointestinal Cancer** | **Moderate** | **Rt PCR** | **No treatment** | **10** |
|  | **SA-COV02** | **F** | **79** | **Lung Cancer** | **Moderate** | **Rt PCR** | **No treatment** | **3** |
|  | **Sand-005** | **M** | **69** | **Clear cell renal cell carcinoma (CCRCC)** | **Severe** | **Cytof**  **Nanostring**  **Rt PCR** | **No treatment**  **(neo-diagnosis)** | **37** |
|  | **Sand-008** | **M** | **70** | **Lung cancer** | **Severe** | **Cytof**  **Multiplex**  **Nanostring**  **Rt PCR** | **No treatment** | **24** |
|  | **Sand-011** | **M** | **69** | **Classical mixed cellularity Hodgkin Lymphoma** | **Severe** | **Cytof**  **Multiplex**  **Nanostring**  **Rt PCR** | **No treatment**  **(neo-diagnosis)** | **55** |
|  | **SA-COV10** | **F** | **65** | **Lung Cancer - Lymphoma** | **Severe** | **Rt PCR** | **No treatment**  **(neo-diagnosis)** | **26** |
|  | **SA-COV25** | **M** | **77** | **Prostate cancer** | **Severe** | **Rt PCR** | **No treatment**  **(resection)** | **3** |
|  | **SA-COV29** | **M** | **81** | **Acute Myeloid Leukemia** | **Severe** | **Rt PCR** | **No treatment** | **30** |
|  | **SA-COV32** | **M** | **74** | **Lung Cancer** | **Severe** | **Rt PCR** | **Gemcitabine treatment before Covid** | **34** |
|  | **Sand-006** | **M** | **74** | **Chronic Lymphatic Leukemia (CLL)** | **Critical** | **Cytof**  **Multiplex**  **Nanostring**  **Rt PCR** | **No treatment**  **(neo-diagnosis)** | **42** |
|  | **SA-COV05** | **M** | **59** | **Lymphoma** | **Critical** | **Rt PCR** | **Radio Therapy Rituximab** | **27** |
|  | **SA-COV07** | **M** | **58** | **Urothelial Cancer** | **Critical** | **Rt PCR** | **Local CHT** | **13** |
| Cancer  (68,9 y  mean age) | **Sand-016** | **F** | **77** | **Lung Cancer** | **N.A.** | **Nanostring**  **Rt PCR** | **No treatment**  **(resection)** | **N.A** |
|  | **Sand-017** | **F** | **65** | **Gastric Cancer** | **N.A.** | **Nanostring**  **Rt PCR** | **No treatment**  **(resection)** | **N.A** |
|  | **Sand-021** | **M** | **65** | **Lung Cancer** | **N.A.** | **Nanostring**  **Rt PCR** | **No treatment**  **(resection)** | **N.A** |
|  | **Sand-025** | **M** | **67** | **Lung Cancer** | **N.A.** | **Nanostring**  **Rt PCR** | **No treatment**  **(resection)** | **N.A** |
|  | **Sand-027** | **F** | **64** | **Endometrial cancer** | **N.A.** | **Nanostring**  **Rt PCR** | **No treatment**  **(resection)** | **N.A** |
|  | **Sand-028** | **M** | **71** | **Lymphoma** | **N.A.** | **Nanostring**  **Rt PCR** | **No treatment**  **(resection)** | **N.A** |
|  | **Sand-029** | **M** | **71** | **Lung Cancer** | **N.A.** | **Nanostring**  **Rt PCR** | **No treatment**  **(resection)** | **N.A** |
|  | **Onco-4** | **M** | **64** | **Clear Cell Renal Cell Carcinoma (CCRCC)** | **N.A.** | **Nanostring**  **Rt PCR** | **Treament** | **N.A** |
|  | **Onco-6** | **M** | **67** | **Lung Cancer** | **N.A.** | **Nanostring**  **Rt PCR** | **No treatment** | **N.A** |
|  | **Onco-7** | **F** | **56** | **Lung Cancer** | **N.A.** | **Nanostring**  **Rt PCR** | **No treatment** | **N.A** |
|  | **Onco-3** | **F** | **72** | **Acute Myeloid Leukemia** | **N.A.** | **Rt PCR** | **No treatment** | **N.A** |
|  | **Onco-5** | **M** | **88** | **Lung Cancer** | **N.A.** | **Rt PCR** | **Immunotherapy** | **N.A** |

**Supplementary Table II**

Antibodies list used to identified the immune cell subsets in the CyTOF analysis.

**Supplementary Table III**

Gene list from the heatmap matched with raw data from Terrier group containing the most deregulated genes in COVID versus cntrl samples.

**Supplementary Table IV**

Cells and Transcription factors list used for the UCSC genome browser ChIP-seq analysis.

**Supplementary Table V**

Binding of IRFs, STATs, BATF, HDACs p300 and E2Fs to the regulatory regions of the 10 genes specifically modulated in COVID-19 cancer compared to cancer patients.

Supplementary Information file is a Word format file containing:

- Supplementary material and methods;
- Supplementary Figure S1,S2, S3, S4, S5, S6, S7;
- Supplementary Table I, II, III, IV, V.
